# Brain structural correlates of schizotypal signs and subclinical schizophrenia nuclear symptoms in healthy individuals

**DOI:** 10.1017/S0033291720002044

**Published:** 2022-01

**Authors:** Tina Meller, Simon Schmitt, Ulrich Ettinger, Phillip Grant, Frederike Stein, Katharina Brosch, Dominik Grotegerd, Katharina Dohm, Susanne Meinert, Katharina Förster, Tim Hahn, Andreas Jansen, Udo Dannlowski, Axel Krug, Tilo Kircher, Igor Nenadić

**Affiliations:** 1 Department of Psychiatry and Psychotherapy, Philipps-Universität Marburg, Rudolf-Bultmann-Str. 8, 35039 Marburg, Germany; 2 Center for Mind, Brain and Behavior (CMBB), Hans-Meerwein-Str. 6, 35032 Marburg, Germany; 3 Department of Psychology, University of Bonn, Kaiser-Karl-Ring 9, 53111 Bonn, Germany; 4 Psychology School, Fresenius University of Applied Sciences, Marienburgstr. 6, 60528 Frankfurt am Main, Germany; 5 Faculty of Life Science Engineering, Technische Hochschule Mittelhessen University of Applied Sciences, Giessen, Germany; 6 Department of Psychiatry and Psychotherapy, Westfälische Wilhelms-Universität Münster, Albert-Schweitzer-Campus 1, Building A9, 48149 Münster, Germany; 7 Core-Facility BrainImaging, Faculty of Medicine, Philipps-Universität, Rudolf-Bultmann-Str. 8, 35039 Marburg, Germany; 8 Marburg University Hospital – UKGM, Rudolf-Bultmann-Str. 8, 35039 Marburg, Germany; 9 Department of Psychiatry and Psychotherapy, University of Bonn, Bonn, Germany

**Keywords:** Brain structure, morphometry, psychotic-like experiences, schizotypy, SCL-90R, surface-based morphometry, voxel-based morphometry

## Abstract

**Background:**

Subclinical psychotic-like experiences (PLE), resembling key symptoms of psychotic disorders, are common throughout the general population and possibly associated with psychosis risk. There is evidence that such symptoms are also associated with structural brain changes.

**Methods:**

In 672 healthy individuals, we assessed PLE and associated distress with the symptom-checklist-90R (SCL-90R) scales ‘schizotypal signs’ (STS) and ‘schizophrenia nuclear symptoms’ (SNS) and analysed associations with voxel- and surfaced-based brain structural parameters derived from structural magnetic resonance imaging at 3 T with CAT12.

**Results:**

For SNS, we found a positive correlation with the volume in the left superior parietal lobule and the precuneus, and a negative correlation with the volume in the right inferior temporal gyrus [*p* < 0.05 cluster-level Family Wise Error (FWE-corrected]. For STS, we found a negative correlation with the volume of the left and right precentral gyrus (*p* < 0.05 cluster-level FWE-corrected). Surface-based analyses did not detect any significant clusters with the chosen statistical threshold of *p* < 0.05. However, in exploratory analyses (*p* < 0.001, uncorrected), we found a positive correlation of SNS with gyrification in the left insula and rostral middle frontal gyrus and of STS with the left precuneus and insula, as well as a negative correlation of STS with gyrification in the left temporal pole.

**Conclusions:**

Our results show that brain structures in areas implicated in schizophrenia are also related to PLE and its associated distress in healthy individuals. This pattern supports a dimensional model of the neural correlates of symptoms of the psychotic spectrum.

## Introduction

Suspiciousness, paranoid thinking, as well as feelings of alienation and isolation are key symptoms of psychotic disorders like schizophrenia. However, it is well-established that reports of psychotic-like experiences (PLE) are also frequently found in the general population, sparking a continuum model of psychosis-proneness (Claridge, 1997). In contrast to the schizophrenia prevalence of ~1% (Simeone, Ward, Rotella, Collins, & Windisch, 2015), psychotic phenomena (e.g. hallucinations) in the absence of the disorder have a lifetime prevalence of ~6–7% in the general population (Linscott & van Os, 2013; McGrath et al., 2015). A considerable ~34% of healthy individuals between the age of 20 and 41 report at least mild psychotic signs (Rössler et al., 2015), in child cohorts even up to >60% (Downs, Cullen, Barragan, & Laurens, 2013).

A continuous phenotypic marker of psychosis-proneness is schizotypy, a multidimensional set of schizophrenia-like personality traits that – similar to the manifest disorder – comprises positive (magical thinking, unusual perceptions and beliefs), negative (introversion, anhedonia), and disorganised (cognitive disorganisation, eccentricity) dimensions (Barrantes-Vidal, Grant, & Kwapil, 2015; Debbané & Barrantes-Vidal, 2015; Grant, 2015). Schizotypy can be conceptualised as a stable, underlying predisposition (Kwapil & Barrantes-Vidal, 2015; Kwapil, Chapman, & Chapman, 1999) for more state-like phenomena like PLE, the (momentary) expression of psychotic experiences in non-clinical populations (Chapman & Chapman, 1980; van Os, Linscott, Myin-Germeys, Delespaul, & Krabbendam, 2009), possibly extending towards ‘ultra-high risk’ states defined for early detection and prevention of transition into clinical states (Schultze-Lutter et al., 2015).

**Fig. 1.**
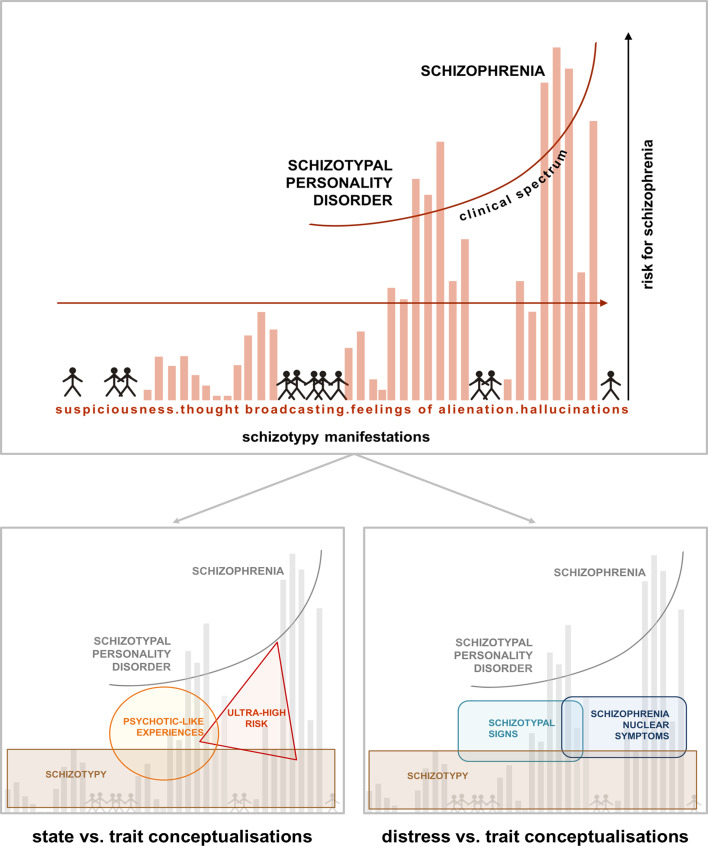
Psychosis continuum model incorporating the STS *v*. SNS dimensions. Upper half [modified from Claridge and Beech (1995)] shows a model of the psychosis continuum where, from the non-clinical towards the clinical parts of the spectrum, symptoms like suspiciousness, thought broadcasting, alienations, and hallucinations increase in intensity in the general population. The model emphasises a dimensional transition across this spectrum, where distress may play an important role in conversion probability. Lower half: within the non-clinical part of the spectrum, different concepts like schizotypy, PLEs and ultra-high risk have been used to capture either trait-like features or state-related clinical aspects (left). The model on the right, depicting STS and SNS, specifically focuses on the distress caused by more trait-related, distress-associated schizotypal personality features (STS) *v*. schizophrenia nuclear symptoms (SNS) closer to the clinical part of the spectrum (right). The overlap of STS and SNS acknowledges the dimensional nature of this alternative approach.

As shown in Fig. 1, the concepts of schizotypy, PLE, and ultra-high risk (UHR) overlap substantially, yet contribute uniquely to conversion prediction, as shown for high schizotypy and UHR (Flückiger et al., 2016; Michel et al., 2019). However, even in high schizotypy, conversion rates into manifest disorders are low – and importantly, rather than the symptom level itself, the level of distress associated with psychotic experiences has greater predictive value (Hanssen, Bak, Bijl, Vollebergh, & Os, 2005a; Hanssen, Krabbendam, De Graaf, Vollebergh, & Van Os, 2005b). At the same time, the association between PLEs and distress seems to be moderated by schizotypy: individuals showing high levels of schizotypy reported more PLEs, but at the same time less distress associated with them, compared to individuals with low trait schizotypy (Kline et al., 2012).

Complementing traditional measures of schizotypal traits, a recent line of research (Rössler et al., 2007) has delineated two dimensions of distress-based assessment of state-like, subclinical psychotic experiences: *schizotypal signs v. schizophrenia nuclear symptoms* (Bakhshaie, Sharifi, & Amini, 2011; Breetvelt et al., 2010; Rössler, Hengartner, Ajdacic-Gross, Haker, & Angst, 2013, 2014; Rössler et al., 2011a, 2011b, 2015; Zhornitsky, Tikàsz, Rizkallah, Chiasson, & Potvin, 2015). Assessed with the widely used symptom-checklist-90R (SCL-90R) symptom checklist (Derogatis, 1977), a questionnaire capturing subjective distress symptoms across several psychological and physical dimensions, they show good internal consistency and validity, and small to medium-sized correlations with traditional schizotypy instruments such as the schizotypal personality questionnaire (Rössler et al., 2007, 2013, 2015). The item wording and response scale are presented in Table 1.

**Table 1.** Items of the SCL-90R scales schizotypal signs and schizophrenia nuclear symptoms with group means and standard deviations (s.d.)

SCL-90R item no.	How much were you distressed by...	Mean (s.d.)
Schizophrenia nuclear symptoms (SNS)		0.11 (0.47)
7	Someone else can control your thoughts.	0.03 (0.21)
16	Hearing voices other people do not hear	0.00 (0.06)
35	Other people being aware of your private thoughts	0.05 (0.25)
62	Having thoughts that are not your own	0.03 (0.22)
Schizotypal signs (STS)		1.68 (2.48)
8	Others are to blame for your troubles	0.15 (0.44)
18	Feeling most people cannot be trusted	0.16 (0.51)
43	Feeling you are watched by others	0.16 (0.45)
68	Having ideas others do not share	0.18 (0.45)
76	Others not giving you proper credit	0.32 (0.62)
77	Feeling lonely even when with people	0.24 (0.58)
83	Feeling people take advantage of you	0.22 (0.52)
88	Never feeling close to another person	0.25 (0.62)

Items are rated (‘How much were you distressed by…’) over the last 4 weeks on a 5 point Likert scale between 0 (‘not at all’), 1 (‘a little bit’), 2 (‘moderately’), 3 (‘quite a bit’), and 4 (‘extremely’) for each item, resulting in scale ranges of 0–16 (*SNS*) and 0–32 (*STS*).

The *schizotypal signs* (STS) scale addresses distress evoked by interpersonal deficiencies, reduced capacity for close relationships, suspiciousness and paranoid ideation, resembling criteria for schizotypal personality disorder and positive and negative dimensions of schizotypy. *Schizophrenia nuclear symptoms* (SNS) assess distress caused by delusions of control, auditory hallucinations, thought-broadcasting and thought-intrusion, representing primarily positive symptoms of the schizophrenia spectrum.

The STS and SNS constructs represent an additional variation of subclinical markers with a special emphasis on distress (see Fig. 1). Although the two scales partially overlap with measures of schizotypy, rather than assessing a general personality disposition, they measure the level of *distress* caused by such experiences in a recent temporal interval. This level may vary across time, as suggested by studies investigating courses of PLE, STS, and SNS (Linscott & van Os, 2013; Rössler et al., 2007). By focussing on symptom distress during the last 4 weeks, STS and SNS close a gap between extremely variable mood and stable personality structure (Franke, 1995). While the temporally restricted assessment implies state-like rather than trait-like character of the scales, longitudinal studies suggest that the variability of state expression can be (partially) attributed to latent, stable traits and shows ‘the trait in action’, possibly representing responses to environmental challenges (Barrantes-Vidal et al., 2015; Rössler et al., 2013).

Growing evidence suggests that both state- (PLEs) and trait-like (schizotypy) expression of such attributes is associated with morphometric variation in certain brain regions. For schizotypy, this includes the precuneus as well as inferior and superior frontal and medial temporal cortical areas (Ettinger et al., 2012; Modinos et al., 2010; Nenadic et al., 2015b; Wang et al., 2015; Wiebels, Waldie, Roberts, & Park, 2016). Similarly, healthy individuals with PLEs as well as individuals at UHR for psychosis show variations in inferior temporal regions, insula and precuneus (Dietsche, Kircher, & Falkenberg, 2017; Fusar-Poli et al., 2011; Nenadic et al., 2015a; van Lutterveld, Diederen, Otte, & Sommer, 2014a; van Lutterveld et al., 2014b). This suggests common neuroanatomical correlates along the psychosis spectrum (Modinos et al., 2010), as those regions also overlap with alterations in schizophrenia (Dietsche et al., 2017).

While previous literature strongly supports volumetric correlates, the association with brain surface morphometry, such as cortical folding or gyrification, still remains largely unknown. Opposed to the high plasticity of volumetric structure, cortical folding, determined during early brain development (Chi, Dooling, & Gilles, 1977), is thought to be a sensitive and stable marker of neurodevelopmental variation (Nenadic, Yotter, Sauer, & Gaser, 2014; Yotter, Nenadic, Ziegler, Thompson, & Gaser, 2011). It might thus indicate neuronal processes long before symptom onset and serve as a surrogate of early neurodevelopmental insult. As altered gyrification patterns within superior temporal, prefrontal, and cingulate cortex have been identified both in psychosis and high risk, the parameter has been suggested as a neurodevelopmental marker for psychosis (Damme et al., 2019; Zuliani et al., 2018).

STS and SNS are psychometrically well-validated, whereas their neuroanatomical correlates still remain unclear. Therefore, the aim of the present study was to test in a large, healthy cohort drawn from the general population the hypothesis that the phenomenologically delineated dimensions *schizotypal signs* and *schizophrenia nuclear symptoms* are associated with volume- and surface-based brain structural correlates similar to those found for schizophrenia and PLEs. Based on current evidence from brain imaging studies, we hypothesised reduced volume in frontal and medial temporal cortical areas and increased volume in the precuneus with increasing symptom load. In addition, we tested the hypothesis that altered gyrification in these regions, as seen in schizophrenia (Spalthoff, Gaser, & Nenadić, 2018), would be related to schizophrenia nuclear symptoms.

## Methods

### Sample

We analysed data from 672 healthy participants [424 female (63.1%), 248 male (36.9%); mean age = 32.51 years, s.d. = 12.23], a subset of the FOR2107 cohort (Kircher et al., 2018), a multi-centre study recruiting from the areas of Marburg and Münster in Germany. All experimental procedures were approved by the local ethics committees of the Medical Schools of the Universities of Marburg and Münster, respectively, in accordance with the current version of the Declaration of Helsinki. We included healthy adults between the age of 18 and 65 years. Exclusion criteria were current or former psychiatric disorders [assessed with SCID-I interviews (Wittchen, Wunderlich, Gruschwitz, & Zaudig, 1997) by trained raters], neurological, or other severe medical disorders, current drug use, verbal IQ<80 [estimated with Multiple Choice Word Test-B (Lehrl, 1995)] as well as common magnetic resonance imaging (MRI) contraindications. All participants volunteered to take part in the study, gave written informed consent and received financial compensation afterwards.

### Assessment of schizotypal signs and schizophrenia nuclear symptoms

All participants completed the German version (Franke, 1995) of the SCL-90R-checklist (Derogatis, 1977) as part of a larger test battery (Kircher et al., 2018) within 14 days of MRI scanning. The SCL-90R is a well-established self-report questionnaire assessing the distress of 90 psychological symptoms across nine dimensions on a 5-point Likert scale, including the dimensions psychoticism and paranoid thinking. Participants were asked to rate symptoms over the past 4 weeks. Based on previous studies (Rössler et al., 2007, 2013, 2015), we computed the sum of the scales ‘schizotypal signs’ (STS; eight items) and ‘schizophrenia nuclear symptoms’ (SNS; four items) that were derived from factor analysis in a large longitudinal cohort study, based on the items of the original SCL-90R scales ‘paranoid ideation’ and ‘psychoticism’ (Rössler et al., 2007). Table 1 shows a list of items for both scales and respective descriptive statistics. SCL-90R has been shown to possess good internal consistency and test-retest-reliability (Derogatis & Cleary, 1977; Schmitz, Hartkamp, & Franke, 2000). In the current sample, the scales show reliability (Cronbach's *α*) of *α* = 0.367 (SNS) and *α* = 0.729 (STS), comparable to data in previous publications: in a large longitudinal cohort study in a German-speaking Swiss sample, Rössler et al. (2013) reported consistency values of *α* = 0.497 to 0.694 (mean 0.595) for SNS, and *α* = 0.800 to 0.869 (mean 0.821) for STS across the multiple time points. The STS and SNS subscales have been derived and validated in epidemiological studies (Rössler et al., 2007, 2015).

### MRI acquisition

High resolution, T1-weighted structural images were acquired on a 3 T MRI system in Marburg (12-channel head matrix Rx-coil; Tim Trio, Siemens, Erlangen, Germany) or Münster (20-channel head matrix Rx-coil; Prisma, Siemens, Erlangen, Germany). At each site, a three-dimensional MPRAGE sequence with slice thickness = 1.0 mm, voxel size = 1.0 × 1.0 × 1.0 mm^3^, field of view FOV = 256 mm and the following parameters in Marburg: acquisition time of TA = 4:26 min, repetition time of TR = 1.9s, echo time TE = 2.26 ms, inversion time TI = 900 ms, 176 slices, flip angle = 7°; and Münster: TA = 4:58 min, TR = 2.13s, TE = 2.28 ms, TI = 900 ms, 192 slices, flip angle = 8°.

### MRI data pre-processing

Pre-processing and voxel-based morphometry (VBM) analyses (Ashburner & Friston, 2000) were executed using the pipeline of the CAT12 toolbox (version 1184, Structural Brain Mapping Group, Jena University Hospital, Jena, Germany) building on SPM12 (Statistical Parametric Mapping, Institute of Neurology, London, UK), running under MatLab (v2017a, The MathWorks, USA) with default parameter settings. Only subjects with at least satisfactory quality measures derived from CAT12 and complete questionnaire data were included in the analyses. Data from both centres were pooled based on extensive quality assurance protocols (Vogelbacher et al., 2018).

For VBM analyses, images were segmented into grey matter, white matter, and cerebrospinal fluid and spatially normalised with the DARTEL algorithm (Ashburner, 2007). All images passed visual quality control (inspection for artefacts and image quality) by experienced researchers (DG, UD) and the homogeneity control implemented in the CAT12 toolbox. Images were smoothed with a Gaussian kernel of 10 mm [full width at half maximum (FWHM)].

We extracted surfaced-based morphometry (SBM) parameters with the CAT12 toolbox, that uses a novel algorithm to extract the cortical surface (Dahnke, Yotter, & Gaser, 2013), allowing to calculate additional information on cortical parameters. We analysed cortical gyrification, based on absolute mean curvature (Luders et al., 2006). Gyrification images were smoothed with a Gaussian kernel of 20 mm (FWHM).

### Statistical analyses

Statistical analyses were conducted using general linear models in CAT12 with a multiple regression design. For both SCL-90R subscales (STS, SNS), separate models were set up to test for associations with grey matter volume (GMV) and gyrification, respectively. To control for the influence of confounding variables, age, sex and site were included in the model as nuisance variables. We also accounted for an Rx coil change after 386 of 445 scans at the Marburg site by including head coil as an additional nuisance variable, as suggested in extensive quality assurance studies (Vogelbacher et al., 2018). In VBM analyses, total intracranial volume was included as an additional covariate. We analysed positive and negative correlations of SCL-90R subscale sum values (STS, SNS) with morphometric parameters in whole-brain analyses. Results were considered significant at *p* < 0.05 cluster-level Family Wise Error (FWE)-corrected for multiple comparisons after an initial cluster-forming threshold of *p* < 0.001.

## Results

### Demographic characteristics

Neither SNS nor STS was correlated with sex (*r* = 0.041, *p* = 0.289; *r* = −0.030, *p* = 0.433, respectively). STS was correlated with age (*r* = 0.077, *p* = 0.046), but SNS was not (*r* = 0.001, *p* = 0.971). SNS and STS showed a significant intercorrelation (*r* = 0.355, *p* = 1.9 × 10^−21^). There were significant differences in age (*p* = 7.3 × 10^−11^) and SNS (*p* = 0.023) between the Marburg and Münster sub-cohorts.

### VBM results

**Fig. 2.**
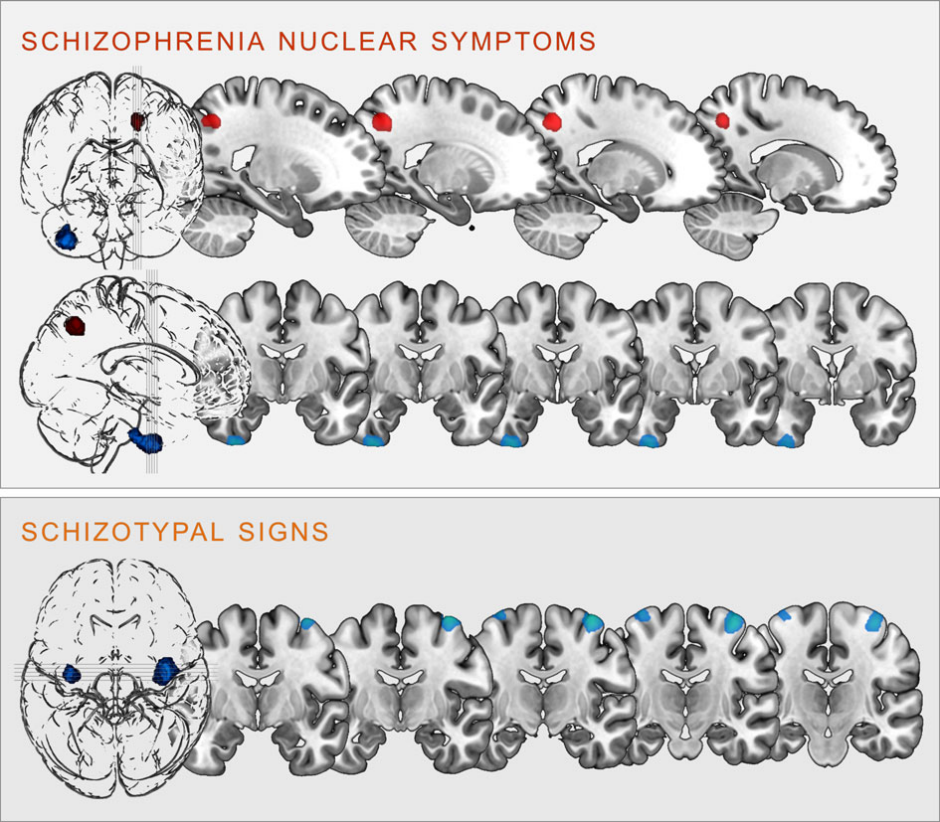
Clusters of significant positive (red) and negative (blue) correlation between grey matter volume and SCL-90R-scales schizophrenia nuclear symptoms (upper panel) and schizotypal signs (lower panel) at *p* < 0.05, cluster-level FWE-corrected (illustration prepared with MRIcroGL; http://www.nitrc.org/projects/mricrogl).

Regression analyses revealed significant correlations between the two scales and clusters in the following brain regions (see Fig. 2):

### Schizophrenia nuclear symptoms

SNS were positively correlated with GMV in the left superior parietal lobe, including parts of the left precuneus (*k* = 406 voxels, *x*/*y*/*z* = −18/−62/45, *T* = 4.56, *p* < 0.001 FWE cluster-level-corrected); and negatively correlated with GMV within the right inferior temporal gyrus (ITG), extending into the right fusiform gyrus (*k* = 915 voxels, *x*/*y*/*z* = 36/−8/-50, *T* = 3.92, *p* = 0.004 FWE cluster-level-corrected).

### Schizotypal signs

STS were negatively correlated bilaterally with the GMV in the right (*k* = 1035, voxels, *x*/*y*/*z* = 38/−10/70, *T* = 4.16) and left (*k* = 298 voxels, *x*/*y*/*z* = −34/−10/75, *T* = 3.86) precentral gyrus (all *p* < 0.001 FWE cluster-level-corrected).

All of these clusters remained significant even after applying an additional Bonferroni correction by adjusting the significance threshold to *p* < 0.0125 (correcting for 4 tests).

### SBM results

#### Schizophrenia nuclear symptoms

**Fig. 3.**
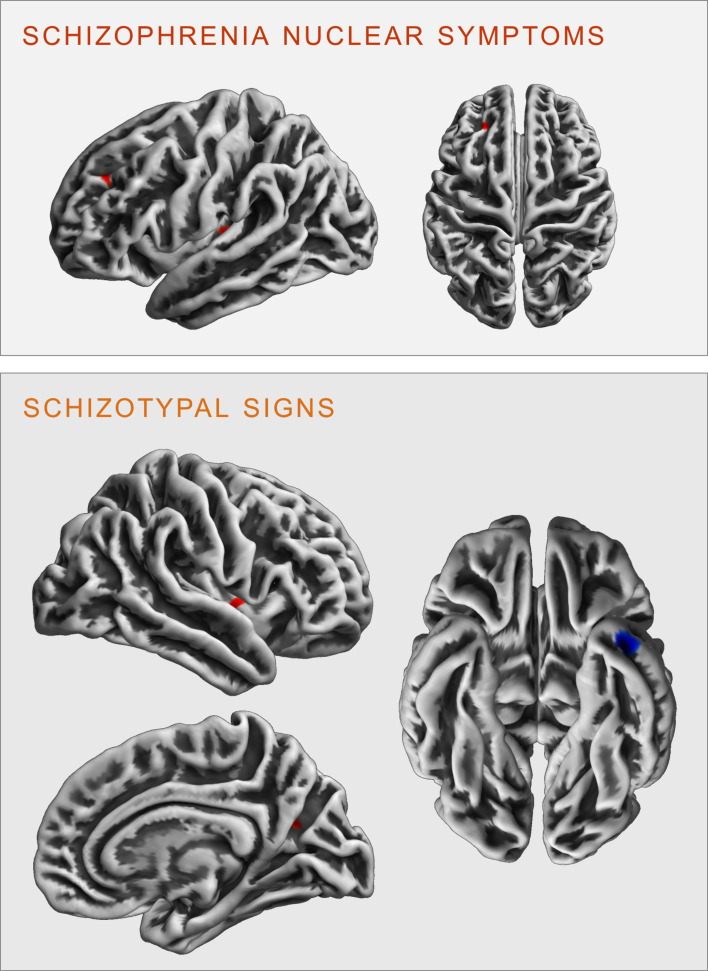
Clusters of significant positive (red) and negative (blue) correlation between gyrification and SCL-90R-scales schizophrenia nuclear symptoms (upper panel) and schizotypal signs (lower panel) revealed in the exploratory analysis at *p* < 0.001 (uncorrected).

There were no significant FWE cluster level-corrected associations of the SNS score with gyrification. However, performing an exploratory analysis (*p* < 0.001 uncorrected), we identified a positive correlation of SNS with gyrification in the left insula (*k* = 27 voxels, *x*/*y*/*z* = −34/−24/5, *T* = 3.32) and the left rostral middle frontal gyrus (*k* = 19 voxels, *x*/*y*/*z* = −23/38/34, *T* = 3.30, see Fig. 3).

### Schizotypal signs

There were no significant FWE cluster level-corrected associations of STS with gyrification. An exploratory analysis (*p* < 0.001 uncorrected), however, revealed that STS was positively correlated with gyrification in the right insula (*k* = 29 voxels, *x*/*y*/*z* = 40/−2/0, *T* = 3.31) and the right precuneus (*k* = 13 voxels, *x*/*y*/*z* = 23/−62/21, *T* = 3.27), as well as negatively correlated with gyrification in the left inferior/middle temporal gyrus (*k* = 42 voxels, *x*/*y*/*z* = −41/3/−37, *T* = 3.78 see Fig. 3).

## Discussion

In the present study, we demonstrated that the level of distress related to PLEs in healthy adults is correlated with brain structural variation, similar to previously reported findings for measures of schizotypy and PLEs. *Schizophrenia nuclear symptoms* (SNS), capturing primarily positive, subpsychotic aspects closer to the clinical spectrum, were positively correlated with precuneus volume and negatively correlated with the volume in the ITG. *Schizotypal signs* (STS), reflecting a milder, personality trait-associated dimension of negative and positive symptoms, were negatively correlated with precentral volume. To date, this is the largest study of the association between brain structure and experiences from the schizotypal spectrum in healthy individuals, including a broader demographic spectrum than most preceding studies. Our results further endorse a dimensional model of neural correlates of schizotypy and psychosis-proneness and highlight the role of emotional appraisal of PLE in the healthy spectrum. This closes an existing gap between psychometrically assessed schizotypy (Claridge, 1997; Grant, 2015) and clinically derived concepts (PLEs, UHR).

The main finding of our study is the disentangling of precuneus volume being linked to distress associated with positive, psychotic-like symptoms (SNS), but not to personality-related STS. This reinforces a spectrum model (shown in Fig. 1) in which sub-psychotic features closer to the clinical spectrum (as captured in SNS) are associated with precuneus variation. Therefore, our results provide evidence for differential brain structural associations of schizotypy and PLEs.

Our finding of the association with precuneus structure suggests its crucial role in mediating a higher risk stage. Precuneus structure has previously been linked to psychometrically assessed schizotypy, indeed this is one of the few findings that has been replicated across several studies (Modinos et al., 2010, 2018; Nenadic et al., 2015b). There is also evidence of links to functional changes: non-clinical individuals with verbal hallucinations show increased precuneus activation (van Lutterveld et al., 2014b), and individuals at clinical high risk for psychosis show a failure to deactivate the precuneus in a working memory task (Falkenberg et al., 2015). These structural and functional findings corroborate the role of the precuneus for symptoms within the psychosis spectrum.

The precuneus, part of the medial parietal cortex, has vast structural and functional connections with multiple brain regions and is thought to be involved in various higher-order cognitive processes (Cavanna & Trimble, 2006; Leech & Sharp, 2014; Zhang & Li, 2012). Its involvement in self-reflection, discrimination of self *v.* others, and cognitive biases like thought-action fusion, reality distortion and self-referential ideas has been shown in studies in obsessive-compulsive disorder and the psychosis spectrum (Cavanna & Trimble, 2006; Jones & Bhattacharya, 2014; Rikandi et al., 2017). Those findings are in line with our own results, linking precuneus volume to thought intrusion and broadcasting, verbal hallucinations and control delusions, as assessed by the SCL-90R *schizophrenia nuclear symptoms* scale.

Concerning our second finding, a negative correlation between SNS and inferior temporal GMV is in line with previously reported cortical thinning in the ITG linked to PLEs, i.e. verbal hallucinations in nonclinical individuals (van Lutterveld et al., 2014b). In one smaller study, volume reductions were also associated with attenuated psychotic symptoms in UHR individuals (Nenadic et al., 2015a). ITG reductions were also part of a pattern distinguishing at-risk individuals with the later conversion from non-converters (Koutsouleris et al., 2010), and have been shown as longitudinal changes following the transition from risk status to psychosis onset (Borgwardt et al., 2008).

While these findings suggest PLEs and clinically derived markers to be associated with ITG structural variations, there is no evidence for such an association with schizotypy. This is consistent with the notion that SNS and STS might tap different parts of the psychosis spectrum. Given the overall low SNS scores in our sample, these findings appear all the more impressive, suggesting similar neuroanatomical correlates of even subtle subclinical variations as shown across the psychosis spectrum. It has to be considered, however, that given the high number of 0 scorers (as commonly seen in healthy population samples) leading to a skewed distribution of the scores, the use of a parametric design – although used in most other previous imaging studies of this kind – has considerable limitations. While large variation of morphometric parameters in subjects with 0 scores might obscure effects (increasing type II errors), we cannot exclude that additional associations might be identified in enriched samples recruiting a higher proportion of high SNS/STS subjects.

In our data, associations with the *schizotypal signs* scale were less prominent and restricted to a negative correlation in a cluster within the right and left precentral gyrus. While precentral gyrus volume decreases are generally associated with motor dysfunctions in schizophrenia (Tanskanen et al., 2010), and one study found a similar pattern in high risk and first episode individuals compared to healthy controls (Chang et al., 2016), it does not generally feature in studies of PLEs in nonclinical individuals. There is, however, evidence for variations in adjacent regions, as reduced grey matter density in the dorsolateral prefrontal cortex has been reported in high *v.* low schizotypy (Wang et al., 2015). Postcentral grey matter reduction was reported in a clinical study with (female) patients with schizotypal personality disorder compared to controls (Koo et al., 2006).

We did not detect associations of the STS scale with areas recently linked to psychometrically assessed schizotypy, such as the precuneus, prefrontal or temporal structures. STS in parts certainly overlaps with commonly used schizotypy measures, still, there are distinct differences: While other measures also often imply distress-proneness, the STS primarily and directly rates the *distress* level rather than that of the symptoms causing it. Also, the blending of positive (e.g. suspiciousness) and negative (e.g. social anhedonia) dimensions rather than distinguishing them may dilute effects. This should be considered as a limitation of the use of STS and SNS, especially with regard to comparability with studies using classical schizotypy measures. This is particularly the case for STS, because positive and negative symptoms as well as positive *v.* negative schizotypy measures might have different neurobiological correlates. Similar to previous studies, the SNS scale showed rather weak internal consistency in our sample, limiting the power to detect associations. While we still detected correlations with a region previously associated with positive symptoms, future studies might use other schizotypy measures with improved psychometric properties.

An important aspect of our finding is the direction of effects, i.e. the positive correlation of SNS and precuneus volume, and a negative correlation with ITG volume. Based on a simplified unidimensional linear association model (continuous volume decreases with increasing symptom load/disease), this might seem counterintuitive. However, comparing our findings to analyses of case-control studies in manifest schizophrenia, these overlap in location and direction of effects, showing both cortical thickening in the precuneus and thinning of ITG (van Erp et al., 2018). Additionally, previous studies have suggested that the relation of subclinical and clinical markers might be modulated by additional variables like cognitive function (Meller, Ettinger, Grant, & Nenadić, 2019), disorder duration (Faget-Agius et al., 2012) or preserved insight (Egashira et al., 2014). Studies in other clinical dimensions, such as subclinical depressive and anxiety symptoms assessed with SCL90-R subscales (Besteher et al., 2019), have suggested that nonlinear functions might describe the relationship. This hypothesis has not yet been tested in larger samples, which might include both major and minor disease variation (i.e. schizophrenia, schizotypal personality disorder, and other schizophrenia spectrum disorders).

Taken together, we show distinct associations with neuroanatomical correlates between SNS and STS, in line with recent findings in schizotypy. The spectrum of PLE towards psychotic symptoms is currently seen as a continuum (Claridge, 1997). This phenomenological continuum, however, is likely not monotonic and unidimensional but falls into several (potentially overlapping) dimensions or facets (Grant et al., 2018), represented by different brain structural (and functional) correlates and networks. By not fully distinguishing between experiences and associated distress, STS and SNS could be viewed as representative of an unidimensional, monotonic, rather than fully dimensional (Claridge, 1997) model, as the distinction between proneness for psychotic (-like) experiences and potential clinical relevance (i.a. schizophrenia-spectrum liability) is at the core of Claridge's conceptualisation of schizotypy (Grant et al., 2018; Schultze-Lutter, Nenadic, & Grant, 2019).

It has been suggested that there are partially distinct susceptibilities to the schizophrenia spectrum (Barrantes-Vidal et al., 2015): While shared genetic variations render certain vulnerabilities to environmental events, other factors might buffer this influence and decrease the impact of schizophrenia risk factors by preserved or increased regional brain volume or function, or stabilised neurotransmitter activity (Siever & Davis, 2004). These models have been mostly explored in schizophrenia *v.* schizotypal personality disorder patients, and only recently extended to the full spectrum including schizotypy in healthy individuals (Meller et al., 2019). Our study further highlights the role of emotional appraisal that might modulate the impact of PLE on brain structure and subclinical *v.* clinical course.

While we did not find an association of the SNS and STS scales with gyrification patterns at corrected threshold levels, the exploratory, uncorrected analysis is of interest due to the associations with the precuneus as well as frontal and temporal regions. So far, there are no cortical folding or gyrification studies analysing associations of those dimensions with developmentally early and rather stable brain structural patterns, so additional studies are warranted. Although the SNS and STS are state-dependent, longitudinal analyses in a large, general population study showed that their variability is largely (75–89%) associated by stable traits (Rössler et al., 2013). This, in line with our results, hints to a possible association of the scales with early, developmental markers, possibly indicating a vulnerability to the psychosis spectrum. In a developmental approach, it has also been argued that an underlying high level of psychometrically assessed schizotypy may constitute an increased liability to state-like subclinical PLE, suggesting an overlap of the partially distinct concepts (Debbané & Barrantes-Vidal, 2015).

Our study addresses the nonclinical spectrum of schizotypal and sub-psychotic symptoms; therefore, as expected, the population shows low symptom loadings and restricted variance. This, however, only reduces statistical power, but should not invalidate the findings (Eysenck, 1952) as it speaks to robustness of the effects.

In conclusion, our results support the notion that structural brain abnormalities in psychosis occur prior to or even independent of the development of full-blown symptoms, may progressively worsen over the course of the illness (Jung, Borgwardt, Fusar-Poli, & Kwon, 2012) and are modulated by emotional appraisal. As such, the phenomenological continuum seems to be reflected in an (at least partial) continuum of neurobiological correlates. It has to be pointed out, however, that the idea of a monotonic linear continuum appears to be overly simplified. Further, the existence of different concepts and phenomenological definitions as well as the use of instruments based thereon might contribute to mixed findings in current research, highlighting the importance of concise conceptualisation (Grant et al., 2018; Lee et al., 2016; Oezgen & Grant, 2018).
